# A Novel MPT‐Driven Necrosis‐Related lncRNA Signature for Prognostic Prediction in Hepatocellular Carcinoma: Validation Using Organoids

**DOI:** 10.1002/cam4.71445

**Published:** 2025-12-26

**Authors:** Yang Liu, Liye Tao, Zefeng Shen, Junhao Zheng, Yali Wang, Meijie Chen, Yangyang Xie, Hongjun Chen, Jingwei Cai, Haoyu Pan, Shihao Li, Renan Jin, Junjie Xu, Xiao Liang

**Affiliations:** ^1^ Key Laboratory of Laparoscopic Technology of Zhejiang Province, Department of General Surgery, Sir Run‐Run Shaw Hospital Zhejiang University School of Medicine Hangzhou China; ^2^ Zhejiang Minimal Invasive Diagnosis and Treatment Technology Research Center of Severe Hepatobiliary Disease, Zhejiang Research and Development Engineering Laboratory of Minimally Invasive Technology and Equipment Hangzhou China; ^3^ Zhejiang University Cancer Center Hangzhou China; ^4^ Liangzhu Laboratory, Zhejiang University Medical Center Hangzhou China; ^5^ Hangzhou Medical College Hangzhou China

**Keywords:** HCC, lncRNAs, MPT‐driven necrosis, organoid, prognostic signature

## Abstract

**Background:**

Mitochondrial permeability transition (MPT)‐driven necrosis, a recently identified form of programmed cell death, significantly influences tumor progression, therapy response, and prognosis. However, research on mitochondrial permeability transition‐driven necrosis‐related long non‐coding ribonucleic acids (MPTDNRlncRNAs) in hepatocellular carcinoma (HCC) remains limited.

**Methods:**

In the current study, we aimed to construct an MPTDNRlncRNA signature to predict survival and classify patients with HCC. RNA sequencing and clinical data were sourced from the Cancer Genome Atlas database, while MPT‐driven necrosis‐linked genes were obtained from the Gene Set Enrichment Analysis database. We identified MPTDNRlncRNAs in HCC tumor tissues and deployed the least absolute shrinkage and selection operator‐Cox analysis to construct a predictive lncRNA signature. Immune cell infiltration variations were analyzed between high‐ and low‐risk subgroups. The MPTDNRlncRNA signature performance was estimated using statistical methodologies, and bioinformatics methods were utilized to investigate functional and pathway differences across risk groups.

**Results:**

A seven‐lncRNA signature specific to HCC was developed, and its predictive accuracy was systematically evaluated using survival analysis, time‐dependent receiver operating characteristic curves, and Cox regression analyses. Correlation analysis demonstrated a strong association between the lncRNA signature and immune cell infiltration, several immune checkpoint targets, and its significant prognostic value for patients with HCC. Additionally, *LINC02313* was recognized as a hub lncRNA in vitro, demonstrating its role in promoting cell proliferation and tumor metastasis. Finally, we validated the function of *LINC02313* using a liver cancer organoid model.

**Conclusion:**

The effective construction of an MPT‐driven necrosis‐related prognostic model highlights its potential to independently predict the prognosis of patients with HCC. These findings not only deepen our understanding of MPT‐driven necrosis but also offer novel theoretical foundations for developing more effective treatment strategies. The gene *LINC02313* has been identified as a promoter of HCC's ability to proliferate and invade, underscoring its potential as a therapeutic target for HCC.

## 1 Introduction

In the global context, primary liver cancer is the fourth leading cause of cancer‐linked mortality, characterized by pronounced histological and biological heterogeneity [1]. Hepatocellular carcinoma (HCC) is the most prevalent form, comprising more than 90% of primary liver malignancies [2]. Recently, despite improvements in the treatment modalities for HCC, including surgery, targeted immunotherapy, and interventional therapy [3], the overall survival (OS) rate remains low due to its low diagnostic efficiency and the occurrence of metastasis [4]. Therefore, it is necessary to develop new biomarkers capable of predicting both survival outcomes and treatment efficacy for improved clinical outcomes.

Mitochondria contribute to several cellular processes, serving as the hub of respiration and exerting substantial influence over cellular metabolism and decisions related to cell fate. Mitochondria can stimulate regulated cell death pathways, particularly those associated with apoptotic or necrotic characteristics [5]. Additionally, mitochondria play a crucial role in processes such as differentiation commitment and the maintenance of the stem cell pluripotent state [6]. Mitochondrial permeability transition (mPT) [7] can induce cellular death, signifying a unique form of cell demise. Accordingly, MPT‐driven necrosis is characterized by significantly elevated mitochondrial inner membrane permeability, high calcium levels, uncoupling oxidative phosphorylation, cellular energy depletion, and cell death by necrosis [8]. Recent evidence indicates that external factors, including dietary intake of substances like monosodium glutamate (MSG), could worsen liver damage and impair mitochondrial balance, thus facilitating cancer development by influencing related signaling pathways [9]. Inducing MPT‐driven necrosis emerges as a viable approach for developing novel cancer treatments. By inducing MPT‐mediated cell death, this method can impede the progression of malignant cells and prevent their transformation into cancerous forms [10].

Long non‐coding RNAs (lncRNAs), a type of non‐coding RNA, frequently exceed 200 nucleotides in length. However, they actively regulate gene expression, affecting cell development, differentiation, and proliferation both transcriptionally and post‐transcriptionally [11]. They significantly contribute to tumor progression and initiation. Abundant evidence supports the involvement of lncRNAs in HCC progression, recurrence, and immunotherapeutic response. These regulatory roles extend across multiple epigenetic pathways, including hypoxia [12], m6A methylation [13], ferroptosis [14], autophagy [15], cuprotosis [16], and energy metabolism [17]. Additionally, recent transcriptomic studies on age‐associated diseases indicate that changes in lncRNA expression may mirror immune system alterations and overall functional decline, reinforcing the idea that immune‐associated lncRNAs can influence tumor progression and treatment outcomes [18]. However, limited studies have explored the contribution of MPT‐driven necrosis‐associated lncRNAs in HCC. Consequently, unraveling their function in HCC holds the potential to enhance our comprehension of its development and lead to innovative therapeutic approaches.

Recently, transcriptomic analyses based on bioinformatics have become effective methods for comprehensively discovering key molecular characteristics and candidate genes in multiple cancers, such as breast and liver cancers [19]. Predicting HCC prognosis has advanced through the development of mitochondrial‐associated gene models [20]. However, the roles and prognostic significance of MPT‐driven necrosis‐associated lncRNAs need to be systematically investigated. In the current study, we developed an MPT‐driven necrosis‐associated lncRNA signature utilizing the Cancer Genome Atlas (TCGA) database for HCC prognosis. The predictive significance of this signature was confirmed, and its correlations with immune cell infiltration and immune checkpoint‐related factors were examined. Furthermore, we investigated the functions of seven essential lncRNAs in HCC, with a particular focus on one pivotal lncRNA, LINC02313. Finally, we conducted thorough investigations both in vitro and on a liver cancer organoid to validate its functionality. Our findings present inaugural evidence of the mechanistic function of *LINC02313* in HCC, offering a potential new therapeutic target for managing HCC.

## 2 Materials and Methods

**FIGURE 1:**
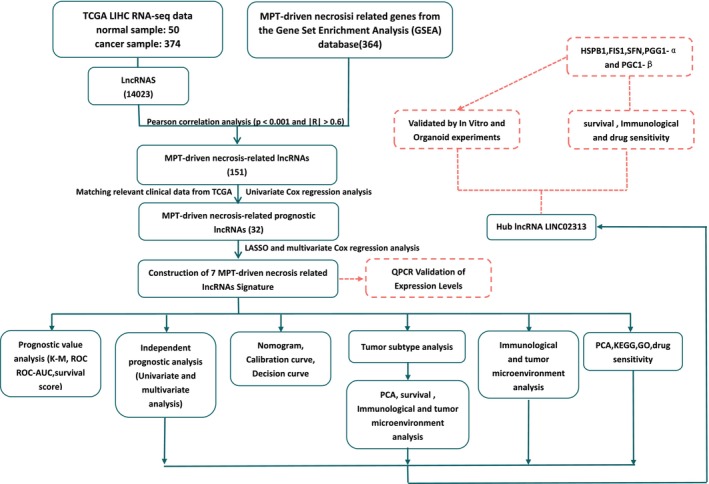
The entire analytical process of the study.

Figure 1 depicts the flow diagram for this study.

### 2.1 Data Collection and Preparation

The RNA‐sequencing for 50 non‐tumor samples, 374 HCC samples, and the associated clinical data for 377 patients with HCC was obtained from TCGA (https://portal.gdc.cancer.gov) on January 15, 2024. The GENCODE lncRNA annotation (Version 22) was utilized to ascertain lncRNAs in TCGA patients with HCC [21]. A total of 364 genes associated with MPT‐driven necrosis, termed MPT‐driven necrosis‐related genes (MPTDNRGs), were identified by screening M17902, M43014, M24979, M13922, M13583, M3873, and M16257.gmt, MPT‐driven necrosis gene sets, in the Gene Set Enrichment Analysis (GSEA) database (GSEA | MSigDB gsea‐msigdb.org) accessed on January 20, 2024.

### 2.2 Determination of MPT‐Driven Necrosis‐Linked lncRNAs and the Construction of the lncRNA‐mRNA Co‐Expression Network

The MPTDNRGs were identified. Subsequently, Pearson correlation analysis was utilized to determine the correlation between lncRNAs and MPTDNRGs (*p* < 0.001, |*R*| > 0.6) to predict MPTDNRlncRNAs. Moreover, Cytoscape software (version 3.6.1, Cytoscape: An Open Source Platform for Complex Network Analysis and Visualization) was deployed to develop a lnRNA‐mRNA co‐expression network utilizing the Pearson correlation coefficients (*p* < 0.001 and |*R*| > 0.6) to determine the connection between MPTDNAs and MPTDNRlncRNAs. Furthermore, a Sankey diagram was constructed to investigate the connections between predictive lncRNAs and MPTDNRNs. This visualization was generated using the R package “ggalluvial” [22].

### 2.3 Creation of MPT‐Driven Necrosis‐Linked lncRNA Signatures

Univariate Cox regression was used to examine patient prognosis‐linked lncRNA genes among the MPT‐driven necrosis‐associated lncRNAs (*p* < 0.05). An MPT‐driven necrosis‐linked lncRNA signature was created utilizing the least absolute shrinkage and selection operator (LASSO) and a multivariate Cox regression model within the training cohort. The multivariate Cox regression coefficient (β) was used to calculate the risk score (RS) with the following equation, incorporating lncRNA expression levels: RS = ∑ᵢₙ Coefficient (lncRNAᵢ) * Expression (lncRNAᵢ). Seven robust lncRNAs were ultimately included in developing the optimal prognostic model [23]. Additionally, patients with HCC were categorized into a low‐risk group (LRG) and a high‐risk group (HRG).

### 2.4 Evaluation of Risk Model Accuracy, Independence, and Nomogram Development

The prognostic signature was meticulously evaluated using many statistical methodologies. Kaplan–Meier (KM) survival analysis was conducted to compare OS between the two cohorts, employing the “survminer” and “survival” R packages along with the log‐rank test. Furthermore, the model's predictive performance was assessed using the “timeROC” R package to calculate the area under the receiver operating characteristic curve [24]. Principal component analysis (PCA) was used to examine the expression pattern distribution between the two groups. Following this, the association between survival outcomes and clinical‐pathological variables was determined using univariate and multivariate Cox regression analyses in all patients with HCC. The accuracy of survival time predictions, including those based on several clinicopathological variables and RSs, was evaluated using ROC curves via the “timeROC” R package [24]. A nomogram was developed by incorporating the lncRNA signature RS with relevant clinical parameters as a quantitative tool for predicting clinical outcomes and supporting clinical decision‐making.

### 2.5 Functional Enrichment Analysis

Differentially expressed genes (DEGs) were filtered using specific risk groups using the criteria: |Log_2_FC| > 1.0, *p* < 0.05. Gene ontology (GO) analysis was conducted to identify biological functions associated with the DEGs, encompassing biological processes (BP), cellular components (CC), and molecular functions (MF). Moreover, pathway analysis of the DEGs was performed using the Kyoto Encyclopedia of Genes and Genomes (KEGG) analysis (KEGG: Kyoto Encyclopedia of Genes and Genomes).

### 2.6 Association of RS With Immune Status and Cancer Stem Cell (CSC) Index

In each HCC sample, the relative abundances of 22 distinct immune cell types were determined using the CIBERSORT methodology. Subsequently, a correlation analysis was conducted to determine the relationship among RS, immune cell compositions, and model‐linked gene expression. Furthermore, ImmuneScore, StromalScore, and ESTIMATEScore for each HCC sample were calculated with the ESTIMATE package, and differential analyses were conducted to compare these scores between the HRG and LRG. Additionally, various immune checkpoint molecules were identified, and differential analyses were performed on their expression levels across HRG and LRG. Finally, a correlation analysis was conducted to determine the association between CSC indices and RS. The CSC index quantifies the similarity between tumor and stem cells, reflecting the degree of stemness on a scale from low (zero) to high (one). The advanced one‐class logistic regression machine learning method was utilized to calculate the CSC index [25].

### 2.7 Mutation and Drug Sensitivity Analyses

The tumor mutational burden (TMB) for each HCC sample was retrieved using the “maftools” R package. Subsequently, a differential analysis of TMB was performed to compare the outcomes between the two risk clusters. Additionally, the IC_50_ values for frequently administered chemotherapeutic drugs were estimated using the “pRRophetic” R package, which predicts chemotherapy response according to tumor gene expression profiles [26]. Differential analyses of the IC_50_ values were then conducted to evaluate variations in therapeutic effects between high‐ and low‐risk clusters.

### 2.8 Consensus Clustering Analysis of MPTDNRlncRNAs

For the HCC subtype analysis, the seven MPTDNRlncRNAs used in model construction were employed. The “ConsensusClusterPlus” R package was deployed to perform consensus clustering analysis. The clustering criteria included maintaining a relatively flat cumulative distribution function curve to avoid a steep transition, ensuring that each subtype contained an adequate sample size, and improving intra‐subtype correlation. Samples were categorized into several subtypes based on the expression profiles of prognostic MPTDNRlncRNAs. Subsequently, survival analysis, tumor microenvironment (TME) analysis, immune cell differential analysis, and other relevant evaluations were conducted to assess the accuracy of HCC subtype clustering.

### 2.9 Cell Culture and Construction of HCC Organoids

The HCC (HepG2 and HCCLM3) and the normal liver (LO2) cell lines (all obtained from the Cell Bank of the Chinese Academy of Sciences, Shanghai, China) were cultivated in DMEM and RPMI 1640 medium, respectively, enriched with 10% fetal bovine serum (FBS) and 1% penicillin–streptomycin (all from Gibco, Carlsbad, California, and USA). Fresh tumor tissue was isolated from HCC patient samples. Under sterile conditions, the tissue was mechanically minced and enzymatically digested using collagenase (abs9444, Absin, Shanghai, China). The resulting cell suspension was filtered to remove large tissue fragments and then collected by centrifugation. The cell pellet was mixed with Matrigel (abs9444, Absin, Shanghai, China), and the resulting mixture was dispensed into a 96‐well plate. After the droplets solidified, an organoid culture medium (abs9444, Absin, Shanghai, China) was added. The HCC cell lines (HepG2 and HCCLM3) were also embedded in Matrigel to establish a cell line‐derived organoid model. Once the droplets had solidified, an organoid culture medium was added. Cultures were incubated at 37°C with 5% CO_2_ and 100% humidity. Tumor tissues and matched peripheral blood samples were obtained from hepatocellular carcinoma patients undergoing surgical resection at Sir Run Run Shaw Hospital. Written informed consent was obtained from all patients prior to the collection and use of both tissue and blood samples. The study was approved by the Ethics Committee of Sir Run Run Shaw Hospital, Zhejiang University School of Medicine (Approval No. 2023‐0728).

### 2.10 Peripheral Blood Mononuclear Cells (PBMCs) and Organoids Co‐Culture

The PBMCs were pre‐cultured for 1 day in an immune cell culture medium containing IL‐2. Patient‐derived organoids were dissociated into single cells and counted. HCC cells and PBMCs were mixed at a ratio of 1:30. The mixed cells were combined with Matrigel and dispensed into a 96‐well plate. After the droplets solidified, 50 μL each of organoid and immune cell culture media were added. The cells were then cultivated for 72 h before performing ATP activity assays.

### 2.11 Quantitative Real‐Time Polymerase Chain Reaction (RT‐qPCR)

Total RNA was extracted from cells using the RNA‐Quick Purification Kit (ES Science, Shanghai, China, cat. RN001), following the manufacturer's protocols. Subsequently, cDNA was synthesized from mRNA using the Yeasen Hifair II 1st Strand cDNA Synthesis SuperMix for quantitative PCR (qPCR; gDNA digester plus, cat. no. 11123ES60). qPCR was performed using the Hieff UNICONQpcr SYBR Green Master Mix (Yeasen, cat. 11198ES30). The standard PCR amplification conditions were as follows: initial denaturation at 95°C for 10 min, followed by 40 cycles of denaturation at 95°C for 10 s, and annealing or extension at 60°C for 30 s. The concluding step encompassed denaturation at 95°C for 15 s, annealing at 60°C for 60 s, and further denaturation at 95°C for 15 s. Gene‐specific primer pairs were custom‐designed and synthesized by Beijing Tsingke Biotech Co. Ltd. (Beijing, China). Primer specificity was verified with Primer‐BLAST against the human RefSeq database. Detailed sequences and corresponding RefSeq accession numbers are listed in Table 1. The relative quantification methodology (2^−ΔΔ*Ct*^) was deployed to ascertain the lncRNAs' relative expression.

**TABLE 1:** The sequences of all primers.

RefSeq ID	Gene	Sequence	
NM_002046.7	GAPDH	Forward primer	CAACGGATTTGGTCGTATTGG
		Reverse primer	TGACGGTGCCATGGAATTT
NR_146544.1	LINC02313	Forward primer	ATGCAGTCATGAGCCCAGTC
		Reverse primer	GAGCTGGGTGACAAATCCACT
NM_016068.3	FIS1	Forward primer	CAAGGAACTGGAGCGGCTCATT
		Reverse primer	GGACACAGCAAGTCCGATGAGT
NM_001540.5	HSPB1	Forward primer	CTGACGGTCAAGACCAAGGATG
		Reverse primer	GTGTATTTCCGCGTGAAGCACC
NM_006142.5	SFN	Forward primer	CTGACGGTCAAGACCAAGGATG
		Reverse primer	GTGTATTTCCGCGTGAAGCACC
NM_133263.4	PGC‐1β	Forward primer	TGAGCAGACCTTGACAGTGGAG
		Reverse primer	GACTATGCTTGATGTCTGGTTTGA
NM_013261.5	PGC‐1α	Forward primer	CCAAAGGATGCGCTCTCGTTCA
		Reverse primer	CGGTGTCTGTAGTGGCTTGACT

### 2.12 CCK‐8 and ATP Assays

HepG2 and LM3 cells, each individually subjected to *LINC02313* knockdown using small interfering RNA (siRNA), along with control cells, were inoculated at a density of 1 × 10^4^ cells/mL in 96‐well plates (100 μL/well). After the cells had fully adhered to the wells, their viability was evaluated at 24 and 48 h. For the viability assay, 10 μL of CCK‐8 reagent (Yeasen, China) was added to each well and incubated at 37°C for 2 h. Moreover, absorbance at 450 nm was measured using a Thermo Fisher enzyme‐labeling instrument (Thermo Fisher, China). Organoids were digested into single cells using collagenase (abs9444, Absin, Shanghai, China) and subjected to *LINC02313* knockdown through siRNA. These cells, along with control organoids, were cultured in a 96‐well plate. To detect ATP, the organoid culture medium was eliminated, and the wells were rinsed with PBS. The ATP detection reagent (CellTiter‐Glo 3D Cell Viability Assay, Promega, USA) was then introduced, and the plate was incubated in the dark for 30 min. Luminescence was measured using a microplate reader (Thermo, Varioskan LUX, China). Afterward, cell proliferation curves were generated using GraphPad Prism (version 9.1.0).

### 2.13 Cell Transfection, Colony Formation, and Invasion Assay

The siRNA was synthesized by RiboBio (Guangzhou, China), and the target sequence *LINC02313* is presented in Table S1. Transfection of these siRNAs into HepG2 and LM3 cell lines was performed utilizing Lipofectamine 3000 (Invitrogen), followed by the manufacturer's protocols, and incubated for 24 h at 37°C. Approximately 1000 control cells, as well as HepG2 and LM3 cells subjected to LINC02313 knockdown, were seeded into 6‐well plates and cultivated for 2 weeks. The cells were subsequently fixed with 10% methanol for 15 min and stained with 0.1% crystal violet for 20 min. The stained colonies were quantified using ImageJ software (version 1.51, National Institutes of Health, USA). For the Transwell assay, approximately 2.5 × 10^5^ cells were seeded into a 24‐well Transwell chamber (Costar, Cambridge, MA) pre‐coated with Matrigel for the invasion assays. The cells were then cultured in DMEM supplemented with 10% FBS for 48 h at 37°C in a humidified atmosphere containing 5% CO_2_. Ultimately, cells that had migrated or invaded to the lower surface of the filter were stained with crystal violet.

### 2.14 Fluorescence‐Activated Cell Sorting Analysis and Measurement of Mitochondrial Membrane Potential (MMP)

For the apoptosis experiment, 3 × 10^5^ cells were suspended in 500 μL of binding buffer, followed by the addition of 5 μL Annexin virtual antigen‐presenting cell (V‐APC) and 5 μL propidium iodide (KeyGEN Biotech, Nanjing, China). The cells were incubated for 10 min, and apoptosis was assessed using flow cytometry. The mitochondrial mass of HCC cells was evaluated employing JC‐1 dye (ThermoFisher Scientific, USA), followed by analysis with a flow cytometer (Becton Dickinson LSR II) or confocal fluorescence microscopy. Fluorescence intensity was then measured and normalized using FlowJo or ImageJ software.

### 2.15 Transmission Electron Microscopy (TEM)

The cells were treated with siNC or si*LINC02313* for 24 h and fixed in 2.5% glutaraldehyde (prepared in phosphate buffer) for 2 h, followed by three rinses with 0.1 M phosphate buffer. They were subsequently fixed in 1% osmium tetroxide for 2 h and rinsed three more times with 0.1 M phosphate buffer. Dehydration was conducted at 4°C through a graded ethanol series. The samples were incubated at room temperature for 4 h in a 2:1 mixture of pure acetone and embedding medium. Ultrathin sections, approximately 50 nm in thickness, were then prepared using an ultramicrotome and examined under a TEM.

### 2.16 Statistical Analysis

All statistical analyses were performed using R software (version 4.2.2). The Wilcoxon test was utilized to compare the proportionate differences in immune‐infiltrated cells within the tumor. Pearson correlation analysis was utilized to investigate the correlations among various variables. The KM methodology was deployed for conducting survival analysis. Significant predictive factors and their independence were assessed using univariate and multivariate Cox regression analyses. The predictive performance of the model for OS was assessed by using the ROC curve. Unless otherwise specified, *p* < 0.05 was deemed statistically significant, with the following annotations: *p < 0.05, **p < 0.01, ***p < 0.001, and ****p < 0.0001.

## 3 Results

### 3.1 Identifying MPT‐Driven Necrosis‐Linked lncRNAs and Constructing the MPTDNRlncRNAs Risk Model for HCC

**FIGURE 2:**
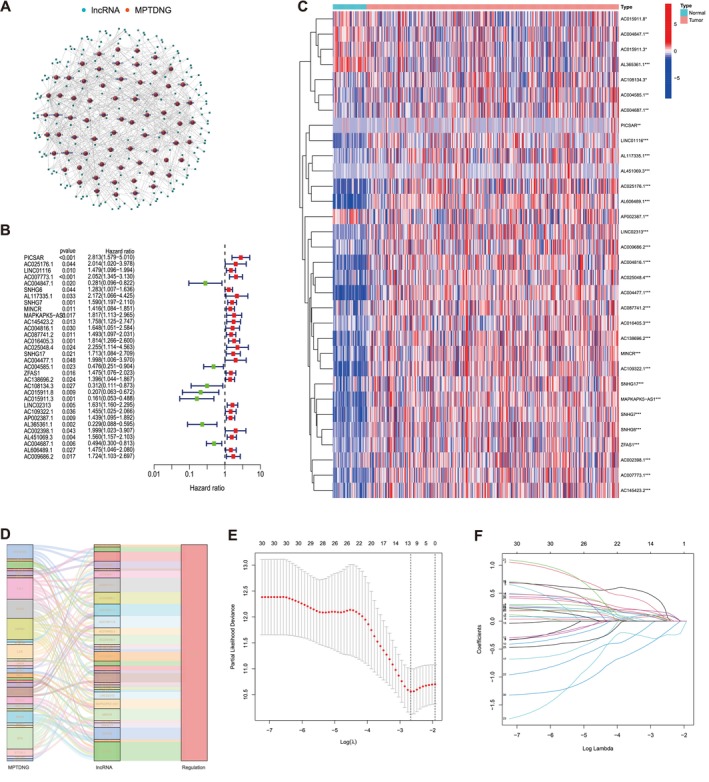
Identification of MPT‐driven necrosis‐related lncRNA prognostic signature in HCC. (A) The network of genes and MPTDNRlncRNAs. (B) The forest plot of prognostic‐related lncRNAs. (C) Heatmap of prognostic‐related lncRNAs. (D) Sankey diagram of MPTDNRGs and MPTDNRlncRNAs. (E) LASSO coefficient profiles of MPTDNRlncRNAs. (F) The partial likelihood deviance with changing of log(λ). *p < 0.05, **p < 0.01, ***p < 0.001, ****p < 0.0001, ns, no significance. HCC, hepatocellular carcinoma; MPTDNRGs, MPT‐driven necrosis‐related genes; MPTDNRlncRNAs, MPT‐driven necrosis‐related lncRNAs.

In the current study, we obtained clinical and transcriptomic data from the TCGA database, including 374 HCC and 50 non‐tumor tissues. We then discovered 151 MPTDNRlncRNAs utilizing Pearson correlation analysis between lncRNAs and 364 MPT‐driven necrosis‐linked genes (Tables S2 and S3). Subsequently, a co‐expression network was constructed to illustrate the relationship between MPTDNRGs and MPTDNRlncRNAs (Figure 2A). Univariate Cox regression analysis revealed that 32 MPTDNRlncRNAs were significantly associated with OS, as depicted in the forest map (Figure 2B,C). Of the 32 lncRNAs, 25 were associated with an unfavorable prognosis for patients with HCC, with higher hazard ratios (HR) indicating an increased risk, while the remaining lncRNAs demonstrated a protective effect. Furthermore, the Sankey diagram demonstrated that all 32 MPTDNRlncRNAs were upregulated in patients with HCC (Figure 2D). The LASSO regression was performed on the 32 MPTDNRlncRNAs to establish the prognostic indicators, guided by multivariate Cox regression. Ultimately, seven MPTDNRlncRNAs (*PICSAR, AC025176.1, AC016405.3, LINC02313, AP002387.1, AC004687.1, and AL451069.3*) were selected to formulate the prognostic model (Figure 2E,F). The cross‐validation and lambda curves are presented in Figure 2E,F. The RS for patients with HCC was calculated using the following formula: RS = *PICSAR* * 0.69439 + *AC025176.1* * 0.95624 + *AC016405.3* * 0.46176 + *LINC02313* * 0.41642 + *AP002387.1* * 0.24750 + *AC004687.1* * (−0.96269) + *AL451069.3* * 0.30881.

### 3.2 The Prognostic Significance of the Novel MPT‐Driven Necrosis‐Linked lncRNAs Signature

**FIGURE 3:**
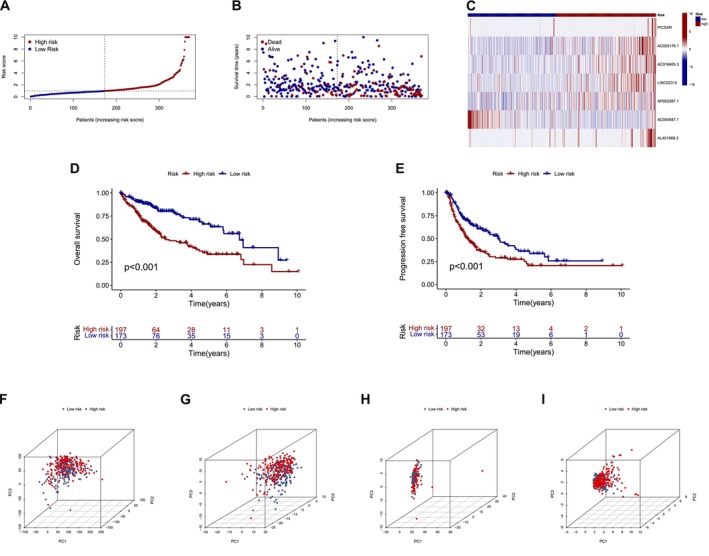
A risk model for outcome prediction. (A) The distribution of the risk scores for each patient. (B) The distributions of the OS status for every patient. (C) The heatmap of five MTPDNRlncRNAs expression. (D) KM curves for the OS of patients in the high‐ and low‐risk groups. (E) KM curves for the progression‐free survival of patients in high‐ and low‐risk groups. (F) PCA of all examined gene expression. (G) PCA of all MPTDNRGs expression. (H) PCA of MTPDNRlncRNAs expression. (I) PCA of the prognostic 7 MPTDNRlncRNAs signature. MPTDNRGs, MPT‐driven necrosis‐related genes; MPTDNRlncRNAs, MPT‐driven necrosis‐related lncRNAs; PCA, principal components analysis.

**FIGURE 4:**
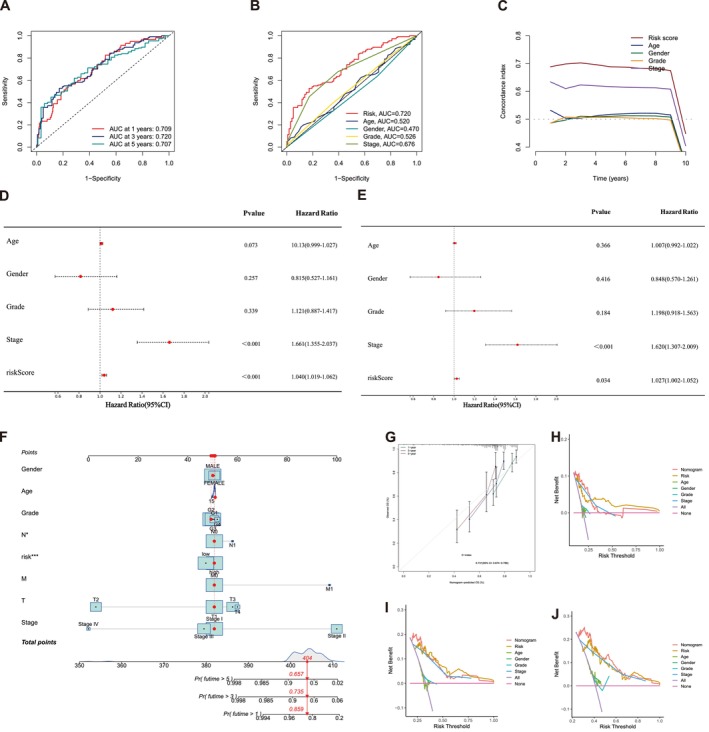
The prognosis value of the novel MPTDNRlncRNAs signature. (A) Accuracy of the risk signature in predicting 1‐, 3‐, and 5‐year ROC curves. (B) The ROC curves of risk score and clinical characteristics. (C) The C‐index curves of risk score and clinical characteristics. (D) The result of univariate Cox regression analysis. (E) The result of multivariate Cox regression analysis. (F) Nomogram for predicting the 0.5‐, 1.0‐, and 1.5‐year OS of patients with HCC. (G) Calibration curve of the nomogram to predict 0.5‐, 1.0‐, and 1.5‐year OS. (H) Decision curve analysis of the nomogram and other risk strategies for predicting 1‐year HCC survival. (I) Decision curve analysis of the nomogram and other risk strategies for predicting 3‐year HCC survival. (J) Decision curve analysis of the nomogram and other risk strategies for predicting 5‐year HCC survival. MPTDNRlncRNAs, MPT‐driven necrosis‐related lncRNAs.

In the current study, we used the RS approach to classify patients with HCC from the TCGA database into LRG and HRG, and we conducted a comparative analysis of these cohorts. The findings revealed that patients in the HRG had significantly shorter survival times (Figure 3A–E), suggesting a poorer prognosis. PCA 3D scatter plots depicted the distribution based on overall gene expressions (Figure 3F), MPTDNRGs expression (Figure 3G), MPTDNRlncRNAs expression (Figure 3H), and the seven‐lncRNA prognostic signature (Figure 3I). Notably, the prognostic signature comprising seven MPTDNRlncRNAs exhibited a clear stratification between the grouped cohorts. To evaluate the predictive performance of the risk score (RS), ROC curves were constructed. The AUC values for the RS were 0.709, 0.720, and 0.707 at 1, 3, and 5 years, respectively (Figure 4A), indicating robust prognostic performance. Additionally, ROC curves were constructed using clinicopathological features, including age, gender, and RS. The RS revealed superior predictive power, with an AUC of 0.720, surpassing individual clinical features. The AUC values for the clinical features were as follows: age (0.520), gender (0.470), grade (0.526), and stage (0.476) (Figure 4B). Additionally, we generated C‐index curves to evaluate the predictive performance of different variables for survival time, with a focus on comparing the RS to other clinical variables. The RS demonstrated superior predictive accuracy, unlike the other clinical characteristics (Figure 4C).

### 3.3 Verification of the Nomogram in HCC

Univariate and multivariate Cox regression analyses were conducted to ascertain whether MPTDNRlncRNAs serve as independent predictive variables for OS in HCC. The univariate Cox regression analysis results demonstrated that stage and RS were significantly associated with OS (Figure 4D, *p* < 0.001). The multivariate Cox regression analysis demonstrated that the RS (*p* < 0.05) could independently function as a predictive marker for OS in patients with HCC (Figure 4E). The findings suggest that the risk model using the seven MPTDNRlncRNAs may function as an independent predictive factor for patients with HCC. Subsequently, we utilized several clinical variables and RSs to develop a nomogram aimed at improving the prediction of 1, 3, and 5‐year survival for patients with HCC, thus improving prognostic accuracy (Figure 4F). The calibration curve was deployed to confirm the nomogram precision (Figure 4G). Furthermore, we performed a decision curve analysis to verify the nomogram accuracy and ascertain the clinical application of the predictive model across various time‐point risk thresholds (Figure 4H–J).

### 3.4 Functional Enrichment Analysis

**FIGURE 5:**
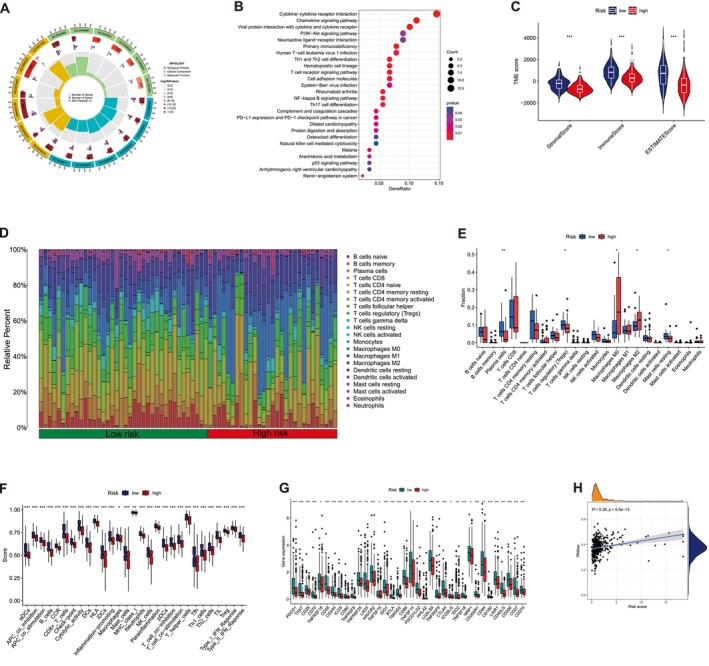
Functional enrichment analysis, immune infiltration landscape, and implications for immunotherapy. (A) Gene ontology (GO) enrichment analysis. (B) KEGG (Kyoto encyclopedia of genes and genomes) enrichment analysis. (C) ESTIMATE algorithm to assess the differences in immune score, stromal score, and estimate score between the two groups. (D, E) CIBERSORT algorithm to evaluate the difference of 22 immune cells between the two groups. (F) ssGSEA algorithm to analyze the differences in immune cells and immune function between the two groups. (G) The difference of common immune checkpoint expression in the risk groups. (H) Correlation analysis between CSC indices and risk scores. *p < 0.05, **p < 0.01, ***p < 0.001, ****p < 0.0001, ns, no significance. BP, biological process; CC, cellular component; MF, molecular function.

In the current study, we conducted GO and KEGG analyses based on 292 DEGs (Table S4) ascertained between both groups to clarify the mechanisms behind the significant differences across the distinct risk groups. The GO analysis findings indicated significant enrichment of DEGs in immune‐related BPs, including antigen binding, immunoglobulin receptor binding, and C − C chemokine binding. Concerning CC, DEGs were significantly enriched in immunoglobulin complexes, the exterior side of the plasma membrane, and the collagen‐rich extracellular matrix. Regarding MF, these DEGs were linked to cell recognition, humoral immune response, and complement activation (Figure 5A).

The KEGG enrichment analysis manifested that these DEGs were primarily enriched in pathways encompassing cytokine–cytokine receptor interaction, chemokine pathway, viral protein interaction with cytokine and cytokine receptor, primary immunodeficiency, Human T‐cell leukemia virus 1 infection, Th1/2 cell differentiation, and T cell receptor pathway, among others (Figure 5B).

### 3.5 Immune Infiltration Landscape Analysis

The TME plays a crucial role in HCC tumor progression and treatment. Accordingly, we investigated the TME across various risk groups employing numerous immune evaluation methodologies. The ESTIMATE findings exhibited that the HRG has demonstrated mitigated immune and estimate scores, unlike those in the LRG (Figure 5C, *p* < 0.05). Moreover, the two groups exhibited significant differences in stromal scores. Consequently, we employed the CIBERSORT method to analyze the types and proportions of 22 immune cells. The outcomes demonstrated discrepancies in the distribution of 22 immune cell types according to the risk model (Figure 5D). The box plot illustrates that the LRG possessed diminished proportions of M0 and M2 macrophages. In contrast, the LRG manifested a greater proportion of plasma, CD4 memory resting T, and resting mast cells (Figure 5E, *p* < 0.05). Subsequently, we utilized the single‐sample gene set enrichment analysis (ssGSEA) to ascertain the discrepancies in the enrichment levels of 16 immune cell‐associated signatures and 13 immune function‐related pathways between the two subgroups. The ssGSEA confirmed significant differences in 29 indicators between LRG and HRG, particularly involving activated dendritic cells, APC co‐inhibition and co‐stimulation, B cells, chemokine receptors, macrophages, follicular helper T cells, and Th2 cells (Figure 5F).

Moreover, we conducted a correlation analysis between RS and 36 immune checkpoint targets to examine the possible function of the MPT‐driven necrosis‐associated lncRNA signature in immune checkpoint blockade (ICB) therapy for HCC. The findings demonstrated significant associations (Figure 5G), indicating that the signature can function as a predictive indicator for the results of ICB treatment in patients with HCC.

### 3.6 Assessment of RS Connection With CSC Index and TMB

**FIGURE 6:**
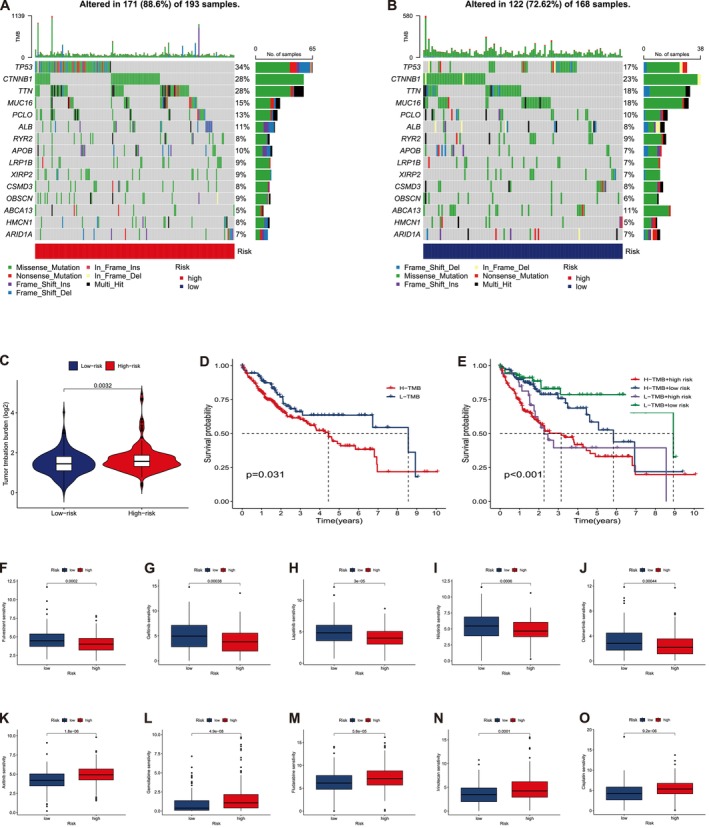
Analysis of somatic mutation landscape and therapeutic sensitivity. (A) Mutation distribution of patients in the high‐risk group. (B) Mutation distribution of patients in the low‐risk group. (C) Differences in TMB scores among different risk groups. (D) KM analysis of OS of patients in different TMB groups. (E) KM analysis of OS among the four groups according to TMB scores and risk scores. (F) Fulvestrant, (G) Gefitinib, (H) Lapatinib, (I) Nilotinib, (J) Osimertinib. (K) Axitinib, (L) Gemcitabine, (M) Fludarabine, (N) Irinotecan, (O) Cisplatin.

The correlation analysis of CSC indexes and RS demonstrated a positive correlation.

(*R* = 0.36, *p* = 5.5e^−13^). The stem cell features of patients with HCC with high RS were more prominent than those of patients with low RS (Figure 5H). Somatic mutations analysis across various risk groups revealed that the LRG exhibited mutations in 122 of 168 samples (72.62%), a lower frequency compared to the HRG, which possessed mutations in 171 of 193 samples (88.60%). The top 15 genes were responsible for these mutations (Figure 6A,B). Analysis of TMB across different risk groups revealed that HRG exhibited a higher TMB than LRG (Figure 6C, *p* = 0.0032). Accordingly, we categorized patients with HCC into low and high TMB groups based on the median TMB score. Patients in the low TMB groups exhibited a significant improvement in OS compared to those in the high TMB group, as indicated by KM analysis (Figure 6D, *p* = 0.031). Moreover, we integrated the TMB score with the RS to predict the prognosis of patients with HCC and ascertain whether the combined score elevated predictive value. Patients with low TMB and risk scores revealed the most favorable OS rates, whereas those with high TMB and risk scores displayed the worst OS outcomes, as indicated by KM analysis (Figure 6E, *p* < 0.001).

### 3.7 Drug Response Analysis

In the current study, we screened prospective pharmacological treatments, considering the significant differences in prognosis, immune microenvironment, and somatic mutations among patients with HCC across several risk groups. Accordingly, we provided precision‐targeted treatment for patients across diverse risk groups. Accordingly, we chose prevalent drugs or compounds to examine the correlation between medication sensitivity and RS. Differential analyses of IC_50_ values between the two risk clusters revealed that the high‐risk cluster exhibited mitigated IC_50_ values of Fulvestrant, Gefitinib, Lapatinib, Nilotinib, and Osimertinib compared with the low‐risk cluster. In contrast, the high‐risk cluster exhibited elevated IC_50_ values for Axitinib, Gemcitabine, Fludarabine, Irinotecan, and Cisplatin compared to the low‐risk cluster. These findings indicate a potential correlation between drug sensitivity and the RS (Figure 6F–O).

### 3.8 Identification of MPTDNRlncRNAs Subtypes in HCC

**FIGURE 7:**
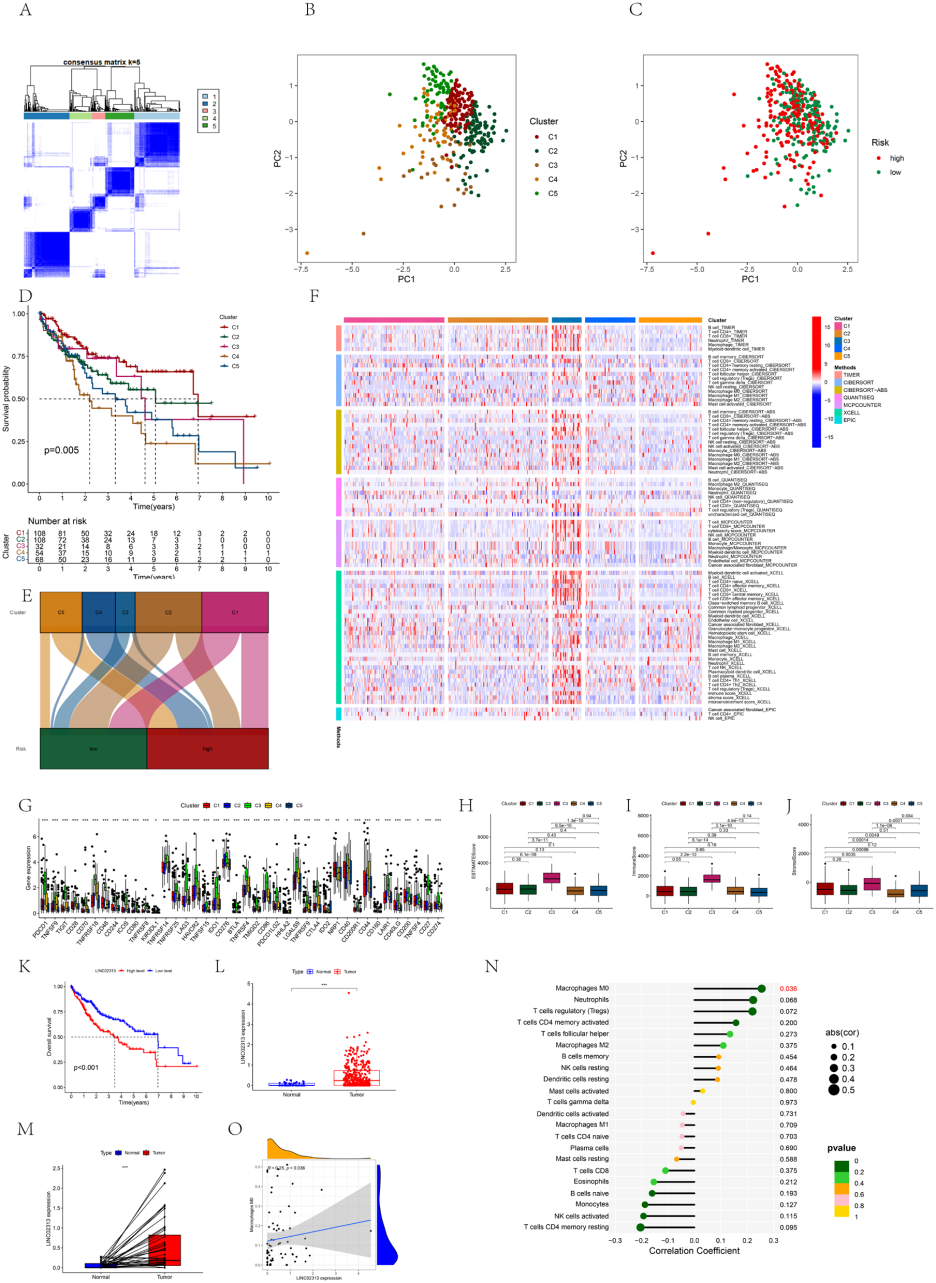
Identification of MPTDNRlncRNA subtypes in HCC and prognostic and immunoregulatory analysis of the hub lncRNA *LINC02313*. (A) Unsupervised clustering analysis of prognostic MPTDNRlncRNAs. Consensus matrix heatmap defining two clusters (*k* = 5) and their correlation area. (B) The PCA analysis based on the prognostic MPTDNRlncRNAs demonstrated that the patients in the different subtypes were distributed in five directions. (C) PCA cluster analysis of the distribution of high and low‐risk groups in each subtype. (D) KM curves for OS of the five MPTDNRlncRNAs subtypes (chi‐square test, *p* = 0.005). (E) The Sankey diagram illustrates the relationships between high and low‐risk groups within each subtype. (F) The heatmap displays differences in the expression levels of immune cells within each subtype. (G) The boxplot illustrates differences in the expression levels of immune checkpoint markers within each subtype. (H–J) ESTIMATE algorithm to assess the differences in estimate score, immune score, and stromal score within each subtype. (K) KM curves for the OS of patients in the high and low expression levels of LINC02313. (L) Overall differential analysis of *LINC02313* expression between cancer and adjacent non‐cancerous tissues. (M) Paired differential analysis of *LINC02313* expression between cancer and adjacent non‐cancerous tissues. (N) Correlation analysis between the expression level of *LINC02313* and immune cells. (O) Correlation analysis between the Expression Level of *LINC02313* and M0 macrophages. *p < 0.05, **p < 0.01, ***p < 0.001, ****p < 0.0001, ns, no significance.

The HCC samples were classified using the consensus clustering methodology according to the seven prognostic MPTDNRlncRNAs expression profiles. The consensus matrix heatmap suggested that the optimal classification methodology is k = 5. As a result, HCC samples were classified into clusters 1 (108 samples), 2 (108 samples), 3 (32 samples), 4 (54 samples), and 5 (68 samples, Figure 7A). The PCA manifested that the transcription profile of MPTDNRlncRNAs across the five subtypes was distinct (Figure 7B,C). Furthermore, KM curves indicated that HCC samples in cluster 1 exhibited superior OS compared to those in the other four clusters, with cluster 4 exhibiting the poorest prognosis (log‐rank test, *p* = 0.005; Figure 7D). The Sankey diagram reveals that most of cluster 4 falls within the HRG (Figure 7E). Moreover, we used the ssGSEA method in each HCC sample to evaluate immune cell infiltration. Significant differences in the relative abundances of certain immune cells were observed among the five subtypes, including MDSCs, CD56+ natural killer cells, macrophages, eosinophils, Th2 cells, mast cells, monocytes, and myeloid dendritic cells. Moreover, cluster 3 demonstrated a significant elevation in the number of most immune cells compared with other subtypes (Figure 7F). The expression levels of PDCD1, TIGIT, LAG3, HAVCR2, and CD27 in cluster 3 were significantly elevated compared to other subgroups for immune checkpoints (Figure 7G). Furthermore, utilizing the estimation of stromal and immune cells in malignant tumors using expression data (ESTIMATE) methodology, we acquired TME scores for each HCC sample, including ImmuneScore, StromalScore, and ESTIMATEScore. ImmuneScore indicates the immune component content, StromalScore denotes the matrix component content, and ESTIMATEScore denotes the sum of them (Figure 7H–J).

### 3.9 Validation Analysis of the Seven MPTDNRlncRNAs in HCC

In the current study, we individually analyzed the overall differences between cancer and adjacent non‐cancer tissues for *PICSAR*, *AC025176.1*, *AC016405.3*, *LINC02313*, *AP002387.1*, *AC004687.1*, *and AL451069.3* to further distinguish the mechanisms of action of the seven MPTDNRlncRNAs in HCC. Besides, we examined the differences between cancer and adjacent non‐cancer tissues for each HCC patient and analyzed the connection between their expression levels and prognosis. The outcomes revealed significant differences in the overall comparison between cancer and adjacent non‐cancer tissues for all seven MPTDNRlncRNAs. Furthermore, the prognostic analysis demonstrated a significant link between the *LINC02313* expression level and prognosis (*p* < 0.001), surpassing the other groups significantly (Figure 7K–M and Figure S1). Interestingly, our analysis of the expression connection between the five subtypes and seven MPTDNRlncRNAs revealed that in the cluster associated with the poorest prognosis, cluster 4, the *LINC02313* expression level was significantly greater than in the other subtypes, whereas in the cluster with the best prognosis, cluster 1, its expression level was the lowest (Table S5). In immune cell correlation analysis, we indicated a positive connection between the *LINC02313* expression levels and M0 (Figure 7N,O). Earlier studies have exhibited that M0 macrophages are significantly involved in liver cancer initiation and progression, and their presence exhibits a negative correlation with the liver cancer patients' prognosis [27]. Consequently, we selected *LINC02313* for further investigation to explore its functionality in HCC. Simultaneously, we analyzed the prognostic relevance of LINC02313 concerning clinical features and immune‐related characteristics (Figure S2A).

### 3.10 qRT‐PCR Identification of *LINC02313* Expression Levels and Its Correlation With MPTDNRGs

**FIGURE 8:**
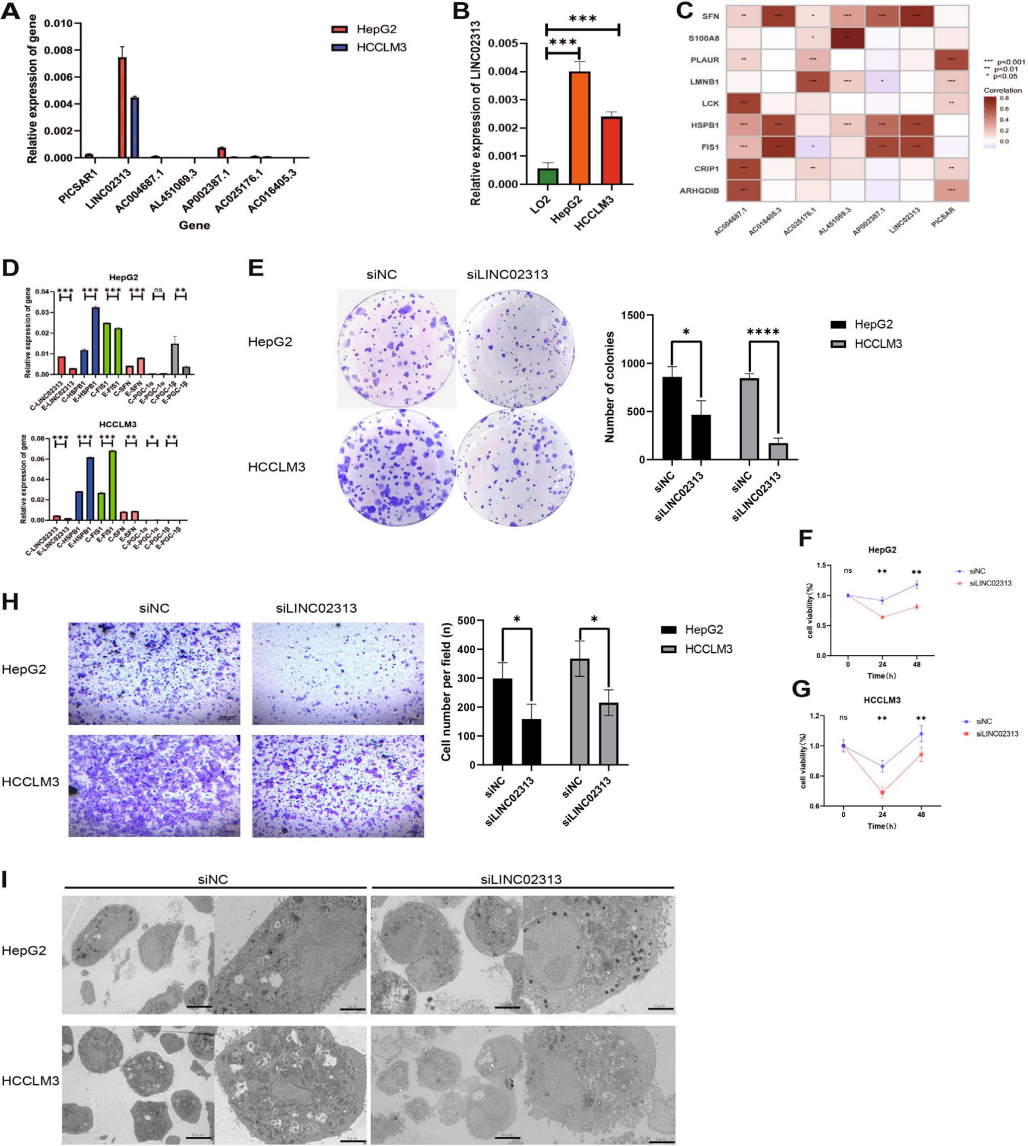
Experimental validation in vitro of the functionality of LINC02313 in HCC. (A) The expression levels of MPTDNRlncRNAs in the subcellular fractions of HCC cells were detected using qRT‐PCR. (B) The relative expression levels of LINC02313 in normal hepatic cells and HCC cells. (C) Correlation analysis of *LINC02313* with MPTDNRNs. (D) Expression levels of related genes before and after silencing *LINC02313* (C‐: control group; E‐: silenced *LINC02313*). (E) Colony formation experiment to assess cell viability. (F) CCK‐8 experiment to evaluate changes in cell viability at 24, 48, and 72 h post‐transfection. (G) Transwell assays were used to detect the invasion of HCC cells. (H) Representative TEM images of mitochondrial morphological changes. Mitochondria in the siNC group remained intact, while those in the si*LINC02313* group changed. Scale, 5 μm. (I) Representative transmission electron microscopy (TEM) images of mitochondrial morphological changes.Mitochondria in the siNC group remained intact, while those in the siLINC02313 group changed.Scale, 5µm. *p < 0.05, **p < 0.01, ***p < 0.001, ****p < 0.0001, ns, no significance.

Additionally, we verified the expression levels of the seven MPTDNRlncRNAsin three HCC cell lines through qPCR. Among the seven lncRNAs, *LINC02313* exhibited the highest expression levels (Figure 8A). Subsequently, we validated *LINC02313* expression levels in normal hepatic (LO2) and HCC (HepG2 and HCCLM3) cell lines. The outcomes exhibited significantly higher *LINC02313* expression in liver cancer cells compared to normal cells (Figure 8B). Subsequently, we conducted correlation analysis and identified three MPTDNRGs to investigate the connection between *LINC02313* and MPTDNRGs (*p* < 0.05, cor > 0.65, Figure 8C), namely *HSPB1, FIS1*, and *Stratifin (SFN)*. Additionally, through a literature review, we identified the common genes PGC1‐α/β, which are known to influence MMP [28, 29]. The RT‐qPCR results revealed that silencing LINC02313 led to varying degrees of expression changes in five other genes, with *HSPB1* exhibiting the most significant upregulation (Figure 8D). Notably, *HSPB1* overexpression has been confirmed to inhibit erastin‐induced ferroptosis, thereby promoting tumor development and metastasis [30].

### 3.11 Silencing *LINC02313* Suppresses the Capability of HCC Cells to Proliferate and Promotes Apoptosis and Metastasis

In the current study, we employed siRNA to silence *LINC02313* expression in HCCLM3 and HepG2 cells to examine its function in HCC. Accordingly, we used CCK‐8 and colony formation assays to assess the impacts of *LINC02313* functional loss on HCC cell proliferation and viability, revealing that the knockdown of *LINC02313* inhibited the proliferation of HepG2 and LM3 cells (Figure 8E–G). Moreover, we conducted Transwell migration assays to examine the contribution of LINC02313 to the invasion of HCC cells. The *LINC02313* knockdown impeded HepG2 and HCCLM3 cell invasion (Figure 8H).

### 3.12 Silencing *LINC02313* Induces Changes in Mitochondrial Membrane Permeability

Accordingly, TEM was utilized to ascertain the *LINC02313* implication on mitochondria. The control group displayed normal mitochondria with an orderly arrangement, distinct membranes, and typical vacuoles. In contrast, after treatment with si*LINC02313*, HepG2 and HCCLM3 cells exhibited reduced and disorganized cristae membranes, increased cristae density, and disruption of both the inner and outer mitochondrial membranes. Most mitochondria revealed shrinkage, and larger vacuoles were also observed (Figure 8I).

### 3.13 Flow Cytometry: Cell Apoptosis and Changes in MMP

**FIGURE 9:**
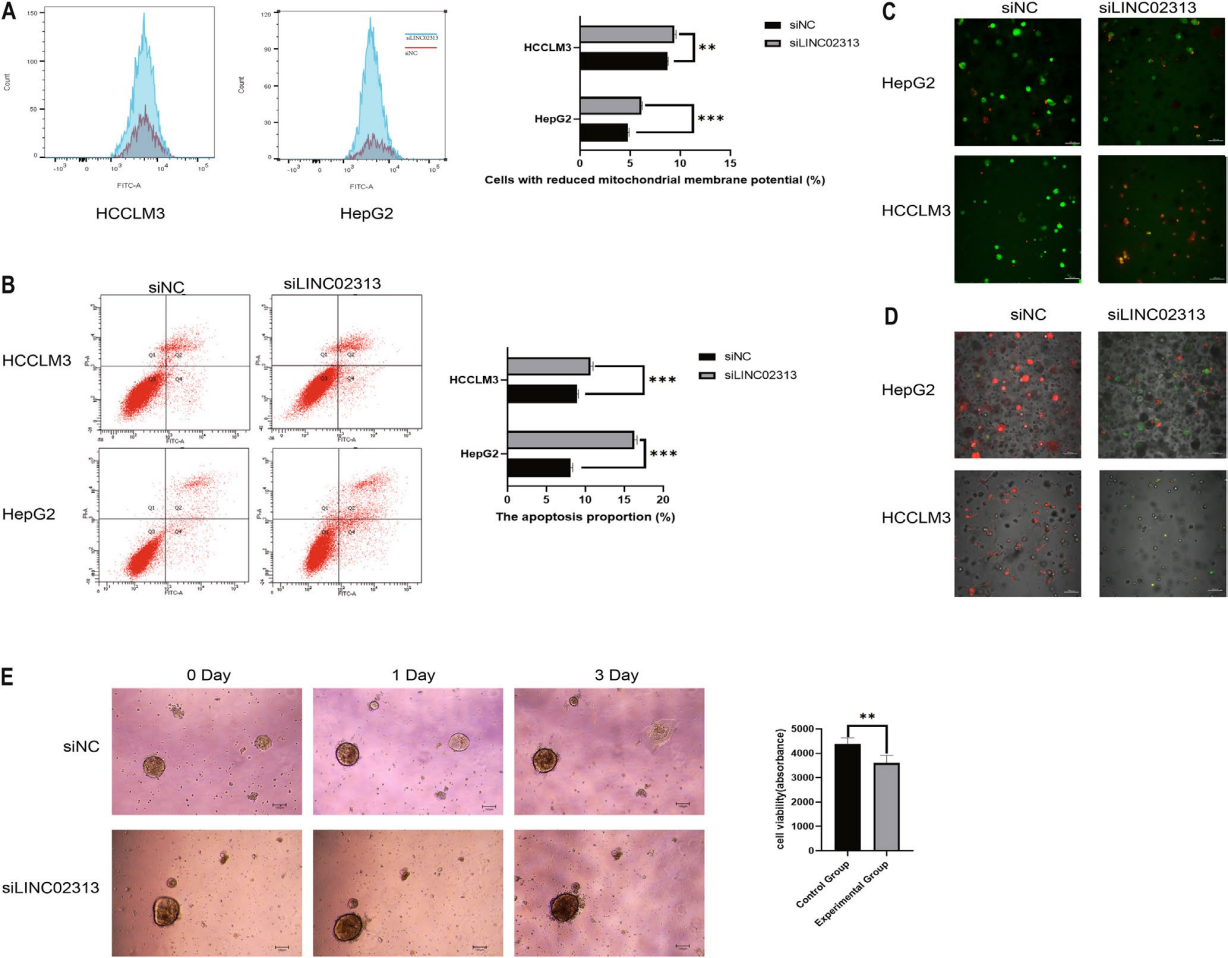
Flow cytometric analysis and organoid validation. (A) Mitochondrial membrane potential changes were assessed using flow cytometry. (B) Cell apoptosis was examined by the flow cytometry. (C) HCC cell line organoid live and dead cell staining. (D) HCC cell line organoids were stained with JC‐1. (E) Co‐culture of PBMCs with organoids and assessment of ATP cellular activity. *p < 0.05, **p < 0.01, ***p < 0.001, ****p < 0.0001, ns, no significance. PBMCs, peripheral blood mononuclear cells.

In the current study, we performed flow cytometry analysis to scrutinize alterations in MMP apoptosis in HCC cell lines (HepG2 and LM3): one with silenced *LINC02313* (experimental group) and the other with unaltered *LINC02313* (control group). The outcomes demonstrated that the experimental group revealed significantly mitigated MMP compared with the control group, along with a substantial increase in the proportion of cells in both early and late apoptosis stages (Figure 9A,B).

### 3.14 Organoid Experiments: Validate *LINC02313* Functionality

The organoid models of HCC cell lines (HepG2 and HCCLM3) were established, followed by live or dead cell staining and JC‐1 staining to determine whether *LINC02313* could function as a therapeutic target for HCC. Fluorescence confocal microscopy revealed a significant increase in cell death (Figure 9C) and a significant reduction in MMP (Figure 9D) after *LINC02313* silencing. Subsequently, patient‐derived HCC organoid models were developed, and *LINC02313* was silenced. Morphological changes and differences in ATP activity were recorded over a 72‐h period. Moreover, we co‐cultured patient‐derived PBMCs with the organoid to simulate the TME. The findings exhibited that, compared with the control group, ATP activity in the *LINC02313*‐knockdown organoids was significantly reduced after 72 h, and there was a greater accumulation of immune cells surrounding the organoids. However, further experiments are required to investigate the interaction between *LINC02313* and immune cells (Figure 9E).

## 4 Discussion

HCC is a prevalent malignancy in the clinical landscape. Despite notable advancements in common therapeutic modalities—including radiotherapy, chemotherapy, and targeted immunotherapy—the overall prognosis for patients with HCC remains suboptimal, accompanied by a diminished quality of life [31]. The mPT is characterized by the sudden loss of the stringent barrier function inherent to the inner mitochondrial membrane (IMM). In the initial homeostasis state, the IMM maintains impermeability through a specialized array of transporters and exchangers, which meticulously regulate the transport of molecules, ions, and metabolites between the matrix and intermembrane space [32]. When left unchecked, mPT has the potential to trigger cellular demise. However, it is crucial to underscore that mPT is a reversible process. Under certain circumstances, a transient flickering of IMM permeability occurs. This phenomenon manifests when the delicate balance inside the cellular milieu is shifted to the negative regulation of mPT [33]. In 2018, MPT‐driven necrosis was officially characterized as a programmed necrotic process, with its mediation attributed to Cyclophilin [34]. mPT now stands as a primary contributor to cellular injury during myocardial infarction [35]. Furthermore, investigations have revealed a noteworthy association between MPT‐driven necrosis and prognostic outcomes in diverse tumors, such as melanoma [36], colorectal cancer [37], and laryngeal squamous cell carcinoma [38]. Nevertheless, the connection between MPTDNRlncRNAs and HCC remains uncertain. To address this gap, we developed a novel prognostic signature for OS incorporating seven MPTDNRlncRNAs. Our investigation explored the multifaceted functions of MPTDNRlncRNAs in HCC, examining various perspectives to unravel their significance in the context of this hepatocellular malignancy.

With the predicted advancements in high‐throughput sequencing technology, numerous public databases and bioinformatics algorithms now serve as valuable tools, offering a scientific foundation for identifying hub genes. These resources facilitate further exploration in cancer research [39]. In the current study, a comprehensive analysis identified 151 MPTDNRlncRNAs through differential expression analysis. Subsequently, a subset of 32 MPTDNRlncRNAs with prognostic implications was ascertained through univariate Cox regression analysis. Afterward, we refined a prognostic signature comprising seven MPTDNRlncRNAs—*PICSAR* [40], *AC025176.1* [41], *AC016405.3* [42], *LINC02313, AP002387.1, AC004687.1* [43], *and AL451069.3* [44]—using LASSO‐Cox regression analysis. All but five of the lncRNAs—*PICSAR, AC025176.1, AC016405.3, AC004687.1, and AL451069.3*—have not been studied. Notably, these five reported lncRNAs exhibit variable expression across different cancer types, significantly involved in both the inhibition and promotion of cancer progression. The remaining two lncRNAs, *LINC02313* and *AP002387.1*, are also closely associated with cancer. The reliability of our model was validated through ROC curve analysis. Moreover, we conducted a KM survival curve analysis, which revealed a substantial correlation between the model and OS. Additionally, univariate and multivariate Cox analyses confirmed that the prognostic signature comprising the seven identified lncRNAs can function as an independent prognostic factor.

The prediction models were utilized to evaluate clinical risks, effectively differentiating between low‐ and high‐risk groups and revealing the practical applicability of the seven MPTDNRlncRNAs. The ROC curve was used to analyze the connection between RS, clinical features, and prognosis, demonstrating that the signature exhibited notable accuracy, with AUC values of 0.709, 0.720, and 0.707 at 1, 3, and 5 years, respectively. Additionally, we constructed a nomogram—a predictive methodology that provides numerical predictions through straightforward calculations. This nomogram, incorporating clinical information and the seven‐MPTDNRlncRNA signature, exhibited strong agreement between predicted and observed OS, underscoring its robust predictive performance.

Moreover, we investigated the prognostic model‐related biological function and pathways, which were developed according to MPTDNRlncRNAs by enrichment analysis. The findings from GO and KEGG analyses revealed a significant correlation between MPTDNRlncRNAs and various immune and intercellular communication pathways. Moreover, we employed GSEA to investigate the potential underlying mechanism linked to a worse HCC prognosis. Our findings indicated that HRG exhibits significantly heightened activities in cell cycle, DNA replication, oocyte meiosis, chromosome organization, and chromosome segregation. These pathways are closely correlated with the replication, proliferation, and invasive characteristics of tumor cells [45]. Nonetheless, immune pathways revealed significant enrichment in the LRG, particularly in pathways encompassing the complement and coagulation cascades. This result indicated that the positive prognosis revealed in the LRG might be linked to immune system activation. Similar phenomena have been previously reported in numerous studies, further supporting these findings. Sun et al. documented that the concurrent administration of FOLFOX4 and ATRA led to a substantial downregulation of proteins associated with the complement and coagulation cascades in patients with HCC who responded effectively to treatment. Coagulation factors F8/9/12 and complement components, C6/8A, indicated downregulation in patients exhibiting complete and partial response, as well as stable disease after therapy [46]. These findings imply that coagulation factors and the complement system may represent potential therapeutic targets, significantly influencing the prognosis of liver cancer.

Tumor immune cell infiltration, pivotal in the tumor immune microenvironment, exerts a significant implication for tumor initiation and progression. The current study revealed that patients with HCC in the LRG exhibited elevated plasma and CD4 memory resting T cell levels and declined M0 and M2 macrophage levels. Previous literature indicates that T cells possess diverse and crucial functions within the immune system, such as promoting B cell class switching, inducing somatic hypermutation in plasma cells, and facilitating memory differentiation. CD4+ T cells contribute to CD8+ T cell proliferation, differentiation, and memory formation. Simultaneously, CD4+ T cells serve as vital effector, cytotoxic, and communication cells. Following stimulation by tumor cells, a T cell subset can form long‐lived memory cells. These memory CD4+ T cells reveal a rapid and efficient response when confronting tumor invasion [47]. Qian et al. revealed that M0 macrophages were associated with the promotion of tumor cell capacity to proliferate and invade. Moreover, Qian et al. revealed that M0 macrophages can enhance tumor cell proliferation and invasion, consistent with our results [48]. Additionally, we performed an analysis to investigate the connection between 7‐MPTDNRlncRNAs, immune cells, and RS. Our findings indicated that the LRG of HCC possessed an increase in most immune cells and immune function scores, reflecting increased immune activity in LRG.

Elevated TMB has been linked to worse survival outcomes and potential inhibition of immune infiltration in HCC [49]. Our findings indicated a significant and positive correlation between risk models constructed by MPTDNRlncRNAs and TMB. The patients with HCC exhibiting high‐risk and ‐TMB scores revealed the worst survival rates and the least favorable prognosis. In contrast, the group of patients with HCC with low‐risk and low‐TMB scores demonstrated heightened survival rates and the most favorable prognosis. This emphasizes the predictive RS superiority in the immunotherapy context for patients with HCC. Immunotherapy has emerged as a revolutionary method, driving significant progress in the treatment of several malignancies and rapidly transforming the clinical oncology landscape [50]. Consequently, we examined the immune checkpoint expression in these two groups and identified that several promising therapeutic checkpoint expressions, including PDCD1, CD86, and LGALS9, were markedly raised in the LRG. This indicates that patients in the LRG may derive substantial benefits from therapies involving anti‐PDCD1, CD86, and LGALS9 antibodies. MPTDNRlncRNAs, particularly *LINC02313*, may have clinical implications beyond prognosis, potentially influencing therapeutic decision‐making. Recent studies show aberrant LINC02313 expression in various cancers, linking it closely with poor prognosis, immune suppression, and treatment resistance. In hepatocellular carcinoma, elevated *LINC02313* correlates with adverse survival outcomes and increased infiltration of M0 macrophages, which contribute to immune evasion and T‐cell exhaustion. Additionally, tumors with high *LINC02313* often present an immunologically “cold” tumor phenotype [51], characterized by reduced PD‐L1 expression and impaired T‐cell infiltration—factors associated with limited response to immune checkpoint inhibitors. Consequently, assessing *LINC02313* expression could help identify patients likely to respond poorly to anti‐PD‐1/PD‐L1 therapies [52]. Conversely, lower expression of *LINC02313* might signify greater immune activity and enhanced responsiveness to immunotherapy. These insights underscore the potential of *LINC02313* not only as a diagnostic marker but also as a predictor for personalized immunotherapeutic interventions in HCC [53].

Furthermore, we assessed the sensitivity of anti‐cancer drugs in patients with HCC across distinct risk groups, offering novel insights for tailored treatment strategies [54]. Our results indicated that Axitinib, Gemcitabine, Fludarabine, Irinotecan, and Cisplatin exhibited heightened efficacy in patients classified under the LRG. Conversely, Fulvestrant, Gefitinib, Lapatinib, Nilotinib, and Osimertinib were more effective in those in the HRG. These findings provide valuable guidance for the clinical management of HCC, facilitating more personalized and effective drug selection for tumor treatment.

Traditional cancer classification is conducted by pathologists based on the histological appearance and anatomical location of the tissue [55]. However, this only partially reflects the heterogeneous nature of cancer. Recent advancements in whole‐genome analysis technologies have enabled researchers to generate extensive genomic data, facilitating the classification of cancer into more homogeneous groups. Clustering analysis of genomic data has been used in studying various cancer subtypes, including leukemia [56], nasopharyngeal carcinoma [57], breast cancer [58], lung cancer [59], and colorectal cancer [60], among others. Accordingly, we delineated five distinct subtypes by leveraging the expression profiles of seven prognostic MPTDNRlncRNAs in HCC samples to refine the classification of HCC. Survival analysis of the five subtypes demonstrated notable differences, with cluster 4 displaying the poorest prognosis and cluster 1 exhibiting the most favorable prognosis. Upon comparing the subtypes, *LINC02313* expression was the lowest in the cluster associated with the best prognosis (cluster 1) and the highest in the cluster linked to the worst prognosis (cluster 4). Additionally, *LINC02313* exhibits upregulation in HCC tissues and connects with a worse prognosis, displaying a positive connection with M0 macrophages. The increased M0 macrophage infiltration has been confirmed to be associated with T‐cell exhaustion and tumor immune evasion [61]. Consequently, we conducted further analysis to delve into the role and mechanisms of *LINC02313* in the HCC initiation and progression.

Eventually, we evaluated the baseline expression level of *LINC02313* in the HCC cell line. Real‐time PCR analysis revealed that, compared to the normal liver cell line LO2, *LINC02313* is significantly overexpressed in two HCC cell lines, suggesting its role in HCC development. Furthermore, when *LINC02313* was knocked down, genes associated with MPT, such as *HSPB1, FIS1*, and *SFN*, were upregulated to exhibit varying degrees, with *HSPB1* exhibiting the most significant increase. According to Arrigo et al., *HSPB1* possesses oncogenic properties stimulating tumor metastasis and closely correlating with resistance to various anti‐cancer drugs [62]. Hao et al. discovered that *FIS1* induces mitochondrial fission and promotes autophagosome formation, participating in the mitochondrial fission‐fusion mechanism and mitochondrial autophagy [63]. An et al. discovered that the elevation of *FIS1* enhances excessive mitochondrial fission, resulting in ATP depletion and heightened mitochondrial reactive oxygen species generation, ultimately culminating in mitochondrial apoptosis [64, 65]. Kim et al. discovered that *SFN* can enhance the development of lung cancer by modulating the nuclear Vps34‐BECN1‐TRAF6 complex to induce autophagy [66]. Research has found that *HSPB1* is closely related to the therapeutic effect of sorafenib [67, 68], and sorafenib, as the first‐line medication for HCC in clinical practice, is often considered the first choice for patients [68]. Therefore, we further validated the drug sensitivity of sorafenib in both *LINC02313* expression groups and indicated that the low expression group exhibits heightened sensitivity to sorafenib (Figure S2F). In vitro experiments revealed that silencing *LINC02313* mitigated the proliferation and invasive potential of HCC cells while enhancing the apoptosis process. In a liver cancer organoid model, we noticed a significant reduction in the activity of tumors with silenced *LINC02313*, validating its capability as a novel therapeutic target. In conclusion, we indicated that *LINC02313* serves as an oncogene in HCC, regulating tumor initiation and development.

To conclude, our study thoroughly assessed the predictive effectiveness and value of MPTDNRlncRNAs in HCC. This encompassed analyzing the pathway enrichment associated with the seven‐MPTDNRlncRNAs signature, exploring immune checkpoint relevance, investigating immune cell infiltration, and evaluating overall prognosis. The findings of the current study contribute to the development of a new, reliable predictive model for patients with HCC. *LINC02313*, identified as a hub gene within the signature, has been acknowledged as an immune‐linked oncogene in HCC, demonstrating its potential as a target for HCC immunotherapy. These outcomes provide promising prospects for improving the treatment of patients with HCC and achieving superior clinical results.

## Author Contributions

**Yang Liu:** methodology, software, writing – original draft. **Liye Tao:** methodology, writing – review and editing. **Zefeng Shen:** conceptualization, formal analysis. **Junhao Zheng:** conceptualization, supervision. **Yali Wang:** conceptualization, data curation. **Meijie Chen:** methodology, project administration. **Yangyang Xie:** methodology, formal analysis. **Hongjun Chen:** investigation, visualization. **Jingwei Cai:** validation. **Haoyu Pan:** validation. **Shihao Li:** validation. **Renan Jin:** validation. **Junjie Xu:** formal analysis. **Xiao Liang:** funding acquisition, writing – review and editing, methodology, resources.

## Funding

Funded by the “Leading Goose” Program of the Zhejiang Provincial Department of Science and Technology (No. 2024C03049), the Major project of Health Science and Technology Program of Zhejiang Province (No. WKJ‐ZJ‐2407), the National Natural Science Foundation of China (No. 82072625), and The Key Project in the Agricultural and Social Development Sector of the Science and Technology Bureau of Hangzhou (No. 20231203A09).

## Ethics Statement

This study was approved by the Ethics Committee of Sir Run Run Shaw Hospital, Zhejiang University School of Medicine (Approval No. 2023‐0728). Written informed consent was obtained from all patients prior to the collection and use of their tissue and blood samples. Additionally, RNA‐seq and clinical data from The Cancer Genome Atlas (TCGA) were utilized in accordance with the data access policies, and no additional ethical approval or patient consent was required for their use. This study does not involve any animal experiments and therefore does not require animal study registration.

## Conflicts of Interest

The authors declare no conflicts of interest.

## Supporting information


**Figure S1:** (A–F) Overall differential analysis of MPTDNRlncRNAs expression between cancer and adjacent non‐cancerous tissues; (G–L). Paired differential analysis of MPTDNRlncRNAs expression between cancer and adjacent Non‐cancerous tissues; *p < 0.05, **p < 0.01, ***p < 0.001, ****p < 0.0001. ns, no significance.

**Figure S2:** (A) Correlation heatmap of clinical pathological features with high and low expression groups of LINC02313; (B) The result of univariate Cox regression analysis for LINC02313; (C) The result of multivariate Cox regression analysis for LINC02313; (D) CIBERSORT algorithm to evaluate the difference of 22 immune cells with high and low expression groups of LINC02313; (E) correlation analysis between LINC02313 and immune checkpoints; (F) drug sensitivity analysis of sorafenib in high and low expression groups of LINC02313; *p < 0.05, **p < 0.01, ***p < 0.001, ****p < 0.0001. ns, no significance.

**Table S1:** The target sequence LINC02313.

**Table S2:** MPT‐driven necrosis‐related genes.

**Table S3:** MPTDNRlncRNAs.

**Table S4:** Differentially expressed genes (DEGs).

**Table S5:** MPTDNRlncRNAs subtypes.

## Data Availability

The data that support the findings of this study are available in the TCGA database at https://portal.gdc.cancer.gov/. These data were derived from the following resources available in the public domain: TCGA database, https://portal.gdc.cancer.gov/—Gene Set Enrichment Analysis (GSEA) database, https://www.gsea‐msigdb.org/gsea/msigdb.
