# Supplementary figures and images for: A Novel MPT‐Driven Necrosis‐Related lncRNA Signature for Prognostic Prediction in Hepatocellular Carcinoma: Validation Using Organoids
Source: Cancer Med. 2025 Dec 26;15(1):e71445. doi: 10.1002/cam4.71445 (PMC12743202; doi:10.1002/cam4.71445)

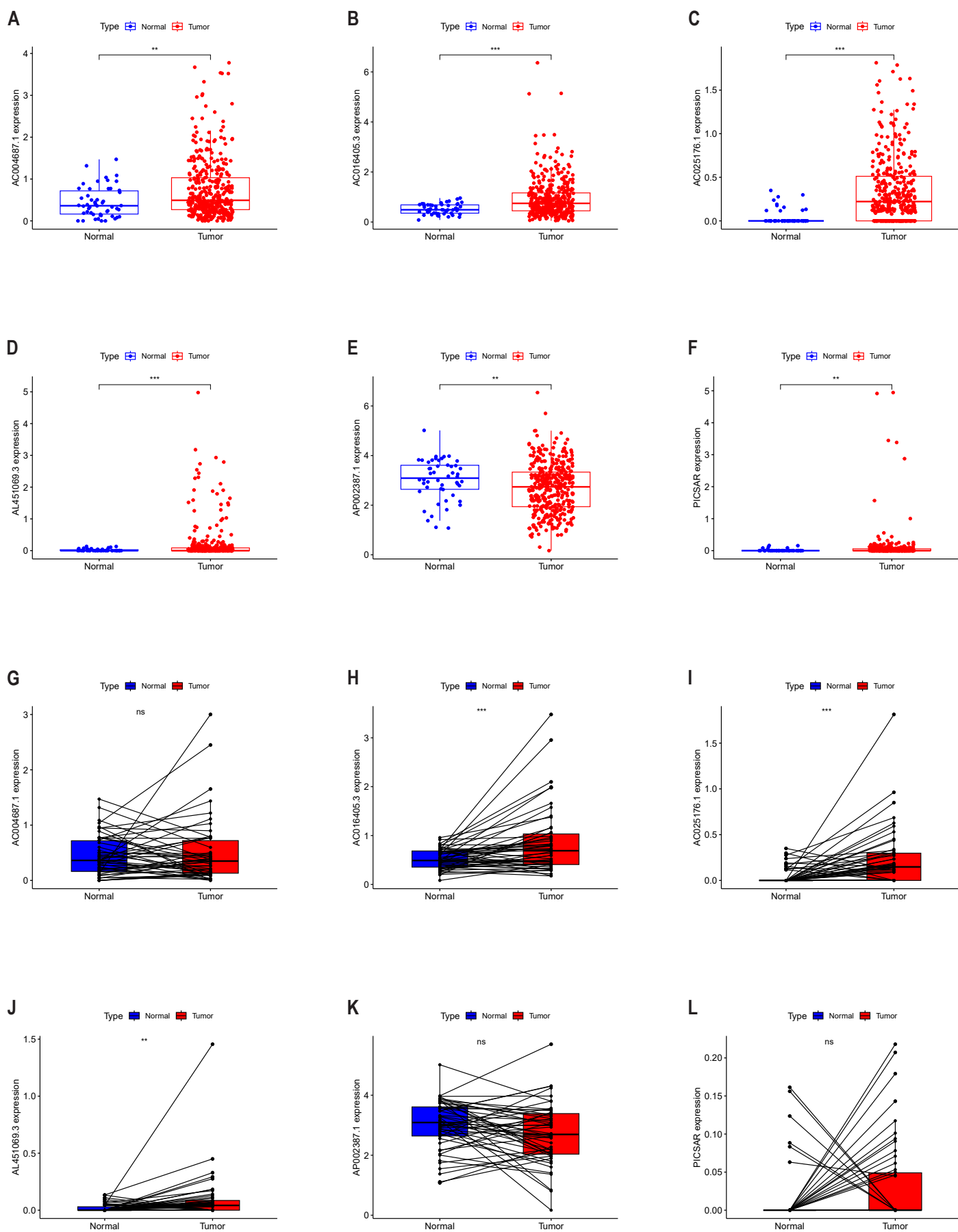

Supplement: Supplementary file 1 — Figure S1: (A–F) Overall differential analysis of MPTDNRlncRNAs expression between cancer and adjacent non‐cancerous tissues; (G–L). Paired differential analysis of MPTDNRlncRNAs expression between cancer and adjacent Non‐cancerous tissues; *p < 0.05, **p < 0.01, ***p < 0.001, ****p < 0.0001. ns, no significance. [file CAM4-15-e71445-s001.pdf]

A

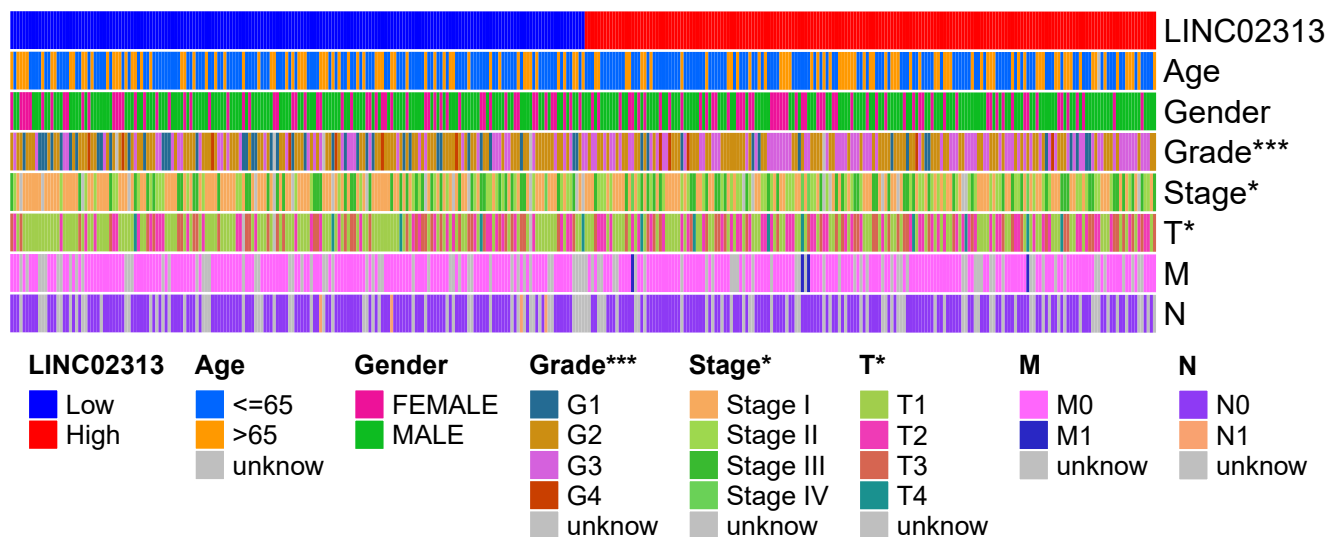

B

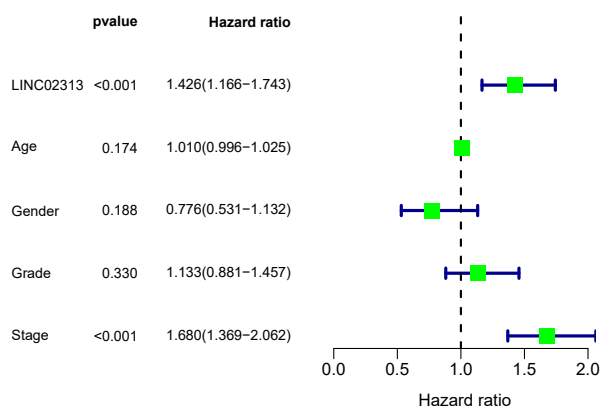

C

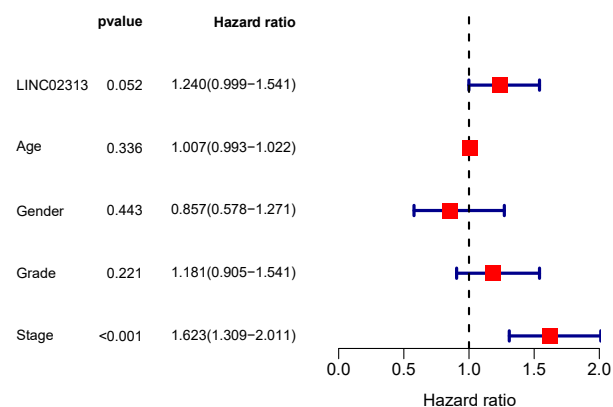

D

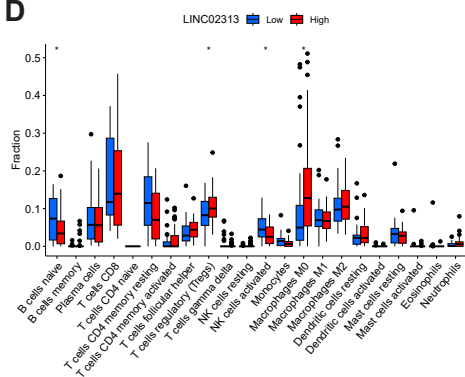

E

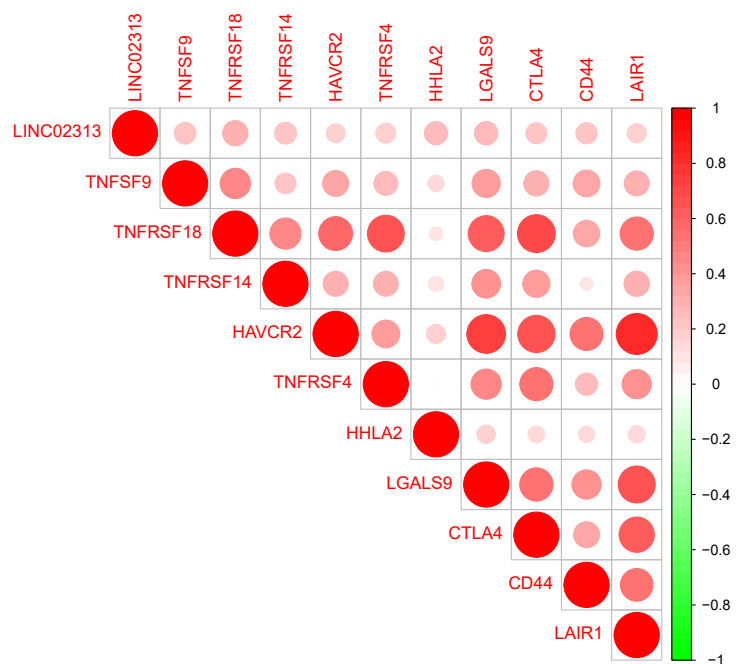

F

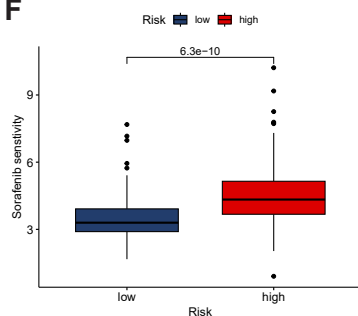

Supplement: Supplementary file 2 — Figure S2: (A) Correlation heatmap of clinical pathological features with high and low expression groups of LINC02313; (B) The result of univariate Cox regression analysis for LINC02313; (C) The result of multivariate Cox regression analysis for LINC02313; (D) CIBERSORT algorithm to evaluate the difference of 22 immune cells with high and low expression groups of LINC02313; (E) correlation analysis between LINC02313 and immune checkpoints; (F) drug sensitivity analysis of sorafenib in high and low expression groups of LINC02313; *p < 0.05, **p < 0.01, ***p < 0.001, ****p < 0.0001. ns, no significance. [file CAM4-15-e71445-s002.pdf]
